# Motor cortex excitability is reduced during freezing of upper limb movement in Parkinson’s disease

**DOI:** 10.1038/s41531-022-00420-w

**Published:** 2022-11-23

**Authors:** Marlene Topka, Marlieke Schneider, Christoph Zrenner, Paolo Belardinelli, Ulf Ziemann, Daniel Weiss

**Affiliations:** 1 Department of Neurodegenerative Diseases, and Hertie Institute for Clinical Brain Research (HIH), University of Tübingen, Tübingen, Germany; 2 Department of Neurology & Stroke, and Hertie Institute for Clinical Brain Research (HIH), University of Tübingen, Tübingen, Germany; 3 Department of Psychiatry, University of Toronto, and Temerty Centre for Therapeutic Brain Intervention, Centre for Addiction and Mental Health, Toronto, Canada; 4 Center for Mind/Brain Sciences - CIMeC, University of Trento, Rovereto (TN), Italy

**Keywords:** Parkinson's disease, Neurological manifestations

## Abstract

Whilst involvement of the motor cortex in the phenomenon of freezing in Parkinson’s disease has been previously suggested, few empiric studies have been conducted to date. We investigated motor cortex (M1) excitability in eleven right-handed Parkinson’s disease patients (aged 69.7 ± 9.6 years, disease duration 11.2 ± 3.9 years, akinesia-rigidity type) with verified gait freezing using a single-pulse transcranial magnetic stimulation (TMS) repetitive finger tapping paradigm. We delivered single TMS pulses at 120% of the active motor threshold at the ‘ascending (contraction)’ and ‘descending (relaxation)’ slope of the tap cycle during i) regular tapping, ii) the transition period of the three taps prior to a freeze and iii) during freezing of upper limb movement. M1 excitability was modulated along the tap cycle with greater motor evoked potentials (MEPs) during ‘ascending’ than ‘descending’. Furthermore, MEPs during the ‘ascending’ phase of regular tapping, but not during the transition period, were greater compared to the MEPs recorded throughout a freeze. Neither force nor EMG activity 10–110 s before the stimulus predicted MEP size. This piloting study suggests that M1 excitability is reduced during freezing and the transition period preceding a freeze. This supports that M1 excitability is critical to freezing in Parkinson’s disease.

## Introduction

Freezing phenomena in idiopathic Parkinson’s disease (PD) represent a significant source of disease-related disability^1–3^. More than 60% of PD patients experience some form of freezing with disease progression, and the incomplete understanding of the pathophysiology and circuit mechanisms limits effective treatment^4,5^. Freezing most commonly occurs while walking (FoG) but also during swallowing^6^, speech^3^, and particularly during repetitive movements of the upper limbs (ULF)^7–9^. Like FoG, ULF significantly impairs daily activities such as handwriting, tooth brushing, typing, or bimanual coordination^1,10,11^. The similar spatiotemporal characteristics of FoG and ULF^12^ prompted research interest in ULF to generate neurophysiological hypotheses on FoG and freezing phenomena in general.

Ultimately, ULF represents the failure to produce an intended movement sequence effectively^2,13,14^. The production of internally generated movement strongly depends on movement planning in frontostriatal projections to M1^15^. The desynchronization of sensorimotor beta-band oscillatory activity during the acceleration phase of a repetitive movement cycle has previously been related to activity across the subthalamic nucleus, the primary motor cortex (M1), and prefrontal areas^8,16^. Furthermore, impaired movement-related modulation of beta-band activity over the sensorimotor area has been associated with freezing behaviour^14,17–19^, and can be observed for up to three taps before a freeze^8^. Combined EEG-TMS studies have established a direct pathophysiological link between oscillatory activity and M1 excitability, showing that M1 excitability was enhanced during beta-band desynchronization but reduced during beta-band synchronization^20,21^.

Here, we studied M1 excitability in PD freezers during regular tapping, transitions, and ULF. Specifically, we investigated the transitions between regular tapping and episodes of ULF using single-pulse TMS time-locked to the tap cycle. Based on the available neurophysiological evidence, we hypothesized that M1 excitability would be modulated throughout the tap cycle with higher excitability during the contraction (ascending) phase and lower excitability during the relaxation (descending) phase. Secondly, we hypothesized that M1 excitability would be reduced during ULF episodes compared to ascending periods of regular tapping. Finally, we anticipated that M1 excitability during the transition period between regular tapping and freezing would show reduced excitability in ascending compared to freezing and lower difference of M1 excitability between ascending and descending.

## Results

### Behavioural findings

On average, all patients completed 15 tapping blocks (*SD* = 5) with a mean tapping frequency of 2.53 Hz (*SD* = 1.29). Within these blocks, we detected 237 freezing episodes across all patients, lasting on average 0.83 s (*SD* = 0.13). In two patients, no TMS pulse coincided with a freezing episode (Table 1) and the data from these patients were excluded from statistical analyses concerning ULF.

**Table 1:** Clinical characteristics of the patient cohort.

Patient	Demographics			Disease					Behavioral Measures						
	Sex	Age	Education	Onset	Duration	Type	Side	LEDD	UPDRS III^1^	N-FOG-Q	M-EDL	nM-EDL	MMSE	TF	n_ULF_
2	m	60	17	2016	4	AR	both	729.5	55	20	20	7	30	2.20	40
3	f	83	9	2006	13	AR	left	296.25		15	18	8	26	1.45	3
4	m	61	15	2012	8	AR	left	364.5		0	2	6	26	3.51	24
5	m	66	12.5	2005	14	AR	right	200	46	16	34	9	29	4.23	16
6	m	70	13	2002	17	AR	left	819	64	22	16	10	30	1.54	50
7	f	78	18	2007	12	AR	both	485	46	21	20	16	30	4.29	2
8	m	63	8	2009	11	AR	right	607	43	19	12	9	27	5.57	26
9	f	76	17	2013	6	AR	both	605	60	20	26	7	29	2.86	41
10	m	53	12.5	2004	15	AR	right	200	37	16	18	11	28	1.96	16
11	f	81	13	2009	11	AR	both	613	63	13	23	10	29	2.88	5
12	m	75	7.5	2009	11	AR	left	1607.6	37	20	13	13	28	2.39	14
*Mean*	-	69.73	14.05	-	11.18	-	-	593.35	46.25	16.63	18.5	9	28.38	2.53	
*SD*	-	9.57	3.3	-	3.87	-	-	395.05	13.26	6.1	8.2	2.91	1.57	1.29	*N* = 237

Age, education, disease duration are displayed in years. *AR* akinetic-rigid Parkinson’s disease, *LEDD* Levodopa Equivalent Daily Dose (mg; see (Schade et al., 2020) for details), *(n)M-EDL* (non) Motor Experiences of Daily Living (nM nonmotor (UPDRS, Part I), *m* motor (UPDRS, Part II)), *MMSE* Mini Mental State Examination, *N-FOG-Q* New-Freezing of Gait-Questionnaire, sex: *m* male, *f* female, *side* disease dominance (more affected side), *n*_*ULF*_ Number of Episodes of Upper Limb Freezing (ULF), TF Mean tapping frequency over all tapping blocks per patient, only full taps were considered (see methods for details), *UPDRS III*^*1*^ Unified Parkinson’s Disease Rating Scale (Motor Examination, Part III), OFF medication.

Overall, 1,067 single pulses were delivered. Of these, 505 were applied during the ascending slope (mean = 27.84% of the tap cycle, *SD* = 9.81), 447 during regular tapping (rTasc; median force = 1.44 N, IQR = 0.67), and 58 during the transition phase (TRANSasc; median force 1.44 N, IQR = 0.58). For the descending slope, 503 pulses were triggered (mean 75.19%, *SD* = 8.06), 425 during regular tapping (rTdesc; median force 1.48 N, IQR = 0.62), and 78 during the transition phase (TRANSdesc; median force=1.51 N, IQR = 0.56). Another 59 pulses coincided with ULF (median force=1.39 N, IQR = 0.76). Finally, 123 of the pulses (36 ascending (29.27%), 80 descending (65%), 7 ULF (5.69%)) did not evoke a MEP (<200µV) and were thus set to value *zero*.

### MEPs during regular tapping, freezes, and transitions

**Fig. 1:**
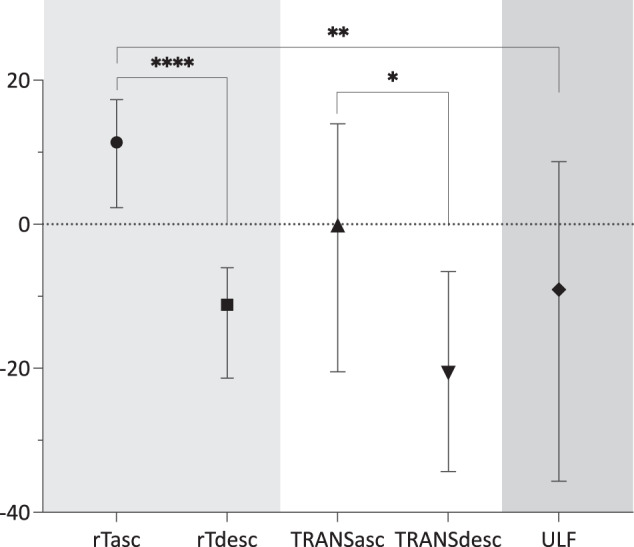
Motor cortex excitability in regular tapping, transitions, and freezing. Overview of all conducted *Mann-Whitney U* tests: we compared normalized MEP sizes (ascending slope vs. descending slope) during regular tapping (rTasc vs. rTdesc), the transition (TRANSasc vs. TRANSdesc), and regular tapping ascending (rTasc) with ULF. MEP sizes were greater at ascending (downward press) compared to descending (upward release) during both regular tapping and transition (see Methods for definition of *transition*). MEPs evoked at rTasc (successful motor output) were also greater than during ULF (unsuccessful motor output), but overall MEP size variability increased when freezing (ULF). X-axis: % change from block average as median ± 95%-confidence interval; Y-axis: motor behavioural state.

We found that during regular tapping, ascending MEPs were greater compared with descending MEPs (*U* = 71402, *P* < 0.001) and greater compared with ULF (*U* = 8904, *P* = 0.007) (Fig. 1). In contrast, MEPs during the descending phase did not differ from ULF (*U* = 11954, *P* = 0.562). During the transition phase, MEPs during the ascending cycle were still greater than for descending (*U* = 1772, *P* = 0.031) but no longer differed from periods of ULF (*U* = 1520, *P* = 0.298).

**Fig. 2:**
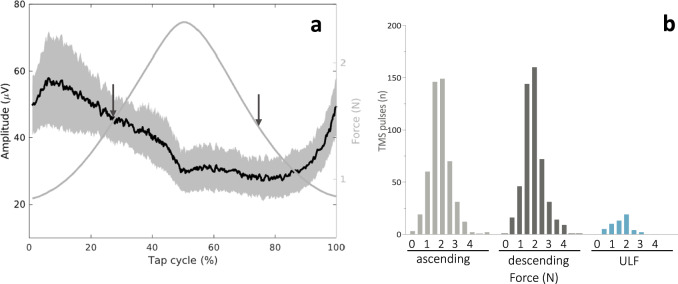
EMG activity and force relative to the tap cycle. **a** Average EMG activity over all single taps of all patients (all data). Black line shows mean EMG activity, grey area shows SEM. A real-time signal processing algorithm (based on Simulink Real-Time Version R2016a, MathWorks Inc., USA) triggered single TMS pulses aimed at 25% tap cycle (=ascending), or 75% tap cycle (=descending), respectively, with an interstimulus interval of >2.5 s. Arrows show the mean percentage of tap cycle at the actual time of TMS (asc: *mean* = 27.84, *SD* = 9.81; desc: *mean* = 75.19, *SD* = 8.06). **b** Histogram of tapping force levels (N) at the time of TMS. There was no significant statistical difference between force levels at the time of TMS. Please note, that ascending and descending each contain both, data from the transition and regular tapping.

As expected, right FDI EMG activity outside the MEPs of both regular and transition time segments was modulated across the tap cycle and was slightly greater for ascending (44.28 ± 6.81µV (*mean* ± *SEM*) compared to descending (28.27 ± 6.09µV; *Z* = −2.045, *P* = 0.041) phases (Fig. 2). However, EMG activity did not differ between ascending and ULF (32.76 ± 5.10µV; *Z* = −1.689, *P* = 0.091) or descending and ULF (*Z* = −1.423; *P* = 0.155) contrasts.

### Secondary analyses to control for potential effects of force and EMG pre-stimulus activity

**Fig. 3:**
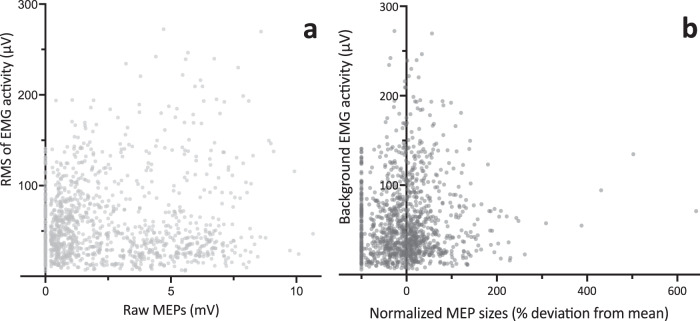
Control analysis - relation of pre-stimulus EMG activity and MEPs. *Spearman’s rho* correlation analysis between background EMG activity 110–10 ms before stimulation (RMS, root mean square) and MEP size. **a**. Correlation with raw MEPs before normalization (*r* = −0.0267, 95% CI −0.0861–0.0329, *p* = 0.3651). **b** Correlation with normalized MEPs (*r* = 0.056, 95% *CI* −0.0032–0.1155, *p* = 0.056).

There was no correlation EMG activity 110–10 ms before a single TMS pulse and MEP size. This held true for both normalized and un-normalized MEP size (Fig. 3). Additionally, force levels at stimulation did not differ between the ascending and descending phases of regular tapping (*U* = 89944, *P* = 0.175), or for transition (ascending vs. descending, U = 2002, *P* = 0.254), or between regular tapping ascending and ULF (*U* = 10178, *P* = 0.19).

Nevertheless, to further exclude any confounding effects of pre-stimulus EMG activity or force on MEP size, we added a linear regression analysis with MEP size as dependent, and behavioural state, EMG activity, and force as independent variables (refer methods for details). Overall, the model showed predictive value on MEP size (R2 = 0.03692, F(4.928)=8.894, *P* = 4.764e-07), as expected. However, this was driven by the differences in the behavioural state between “regular tapping ascending” vs. “descending” (*P* < 0.001) and “regular tapping ascending” vs. “freezing” (*P* = 0.0374). However, neither EMG activity (*P* = 0.511) nor force (*P* = 0.883) were predictive in this model.

## Discussion

We used adaptive single-pulse TMS to study M1 excitability in PD freezers during the ascending (contraction) and descending (relaxation) cycles of regular finger tapping, the transition period, and actual freezing episodes. As expected, cortical excitability fluctuations in M1 were preserved during regular tapping and transitions depending on the movement phase. However, M1 excitability was reduced during freezing compared to the ascending phase of regular tapping, despite the intention to move. During ascending transition taps, M1 excitability was no longer increased compared to freezing and pointed to a premonitory abnormality of M1 in increasing excitability.

In our experiment, background EMG was slightly higher comparing the ascending to the descending slope of tapping, whereas the force levels did not differ between conditions^22^. In control analysis, we did not find a correlation of background EMG with MEP size, nor did we find predictive value of force or EMG background activity when we included these variables to a multiple regression model. However, the effect of behavioural state (regular tapping ascending / descending, freezing) persisted. Thus, it is unlikely that background EMG or force explained our findings^23^. Instead, phase-specific modulation of corticospinal responsiveness has been observed during lower^24^ and upper limb cyclic movements^23^ in younger, healthy subjects, and our findings support that such excitability fluctuations aligned to the movement cycle are preserved in PD freezers. However, PD freezers were impaired when increasing cortical excitability during the freeze and the transition period. In the light of previous research, the insufficient increase of M1 excitability of the Parkinsonian M1 does not necessarily constitute a general abnormality but rather emerges during voluntary movement. Whereas the PD resting motor thresholds were found to be mostly similar to those reported in healthy controls^25^, the Parkinsonian M1 has shown reduced cortical excitability increases during isometric contraction and in reaction time tasks. This was reflected by flattened input-output curves in incremental isometric contractions^26^, and by abnormalities in pre-movement facilitation in a reaction time motor task, such that the Parkinsonian M1 showed an earlier but lower excitability increase prior to externally cued voluntary movements^27,28^. Our findings suggest that the freezing phenomenon may critically depend on such rapid and sufficient adjustments of cortical excitability to stabilize repetitive movement sequences. In turn, failure of these excitability adjustments – as observed during the transition period – may give rise to freezing, which could represent a generalized pathophysiologic mechanism common to the symptoms observed in upper and lower limbs and speech. Indeed, motor deterioration in our experiment became first kinematically visible during freezing but was neurophysiologically measurable in the transition phase already, when cortical excitability decreased. This may have been related to increasing performance difficulties and reflect a “timing problem” when modulating M1 excitability over the tap cycle. In this sense, increasing demands on motor performance such as fatigue or high tapping speed^9^ might have deranged the physiological time course of excitability modulations and preparation of M1 to increase excitability when needed^16^.

In general, M1 excitability and successful motor output rely heavily on afferent inputs from diverse cortical and subcortical areas to M1^29^. Particularly the generation of self-initiated sequential (finger) movements – movement most susceptible to freezing – depends on intact signal transmission from the frontoparietal executive control network^30^ and the BG-thalamic-motor loop, involving the supplementary motor area, M1, putamen, globus pallidus, and ventrolateral thalamus^31^. In PD, reduced effective connectivity between these areas and the loss of automaticity^32^ lead to compensatory over-reliance on the cognitive striatum for effective movement production. The competition for limited processing resources with increasing motor task difficulty is then believed to cause transient striatal dysfunction and STN overactivity, ultimately resulting in freezing^33^.

As such, our observations are compatible with existing pathophysiological models of freezing that have postulated a ‘dysexecutive’ communication failure between frontostriatal and primary motor areas with a paroxysmal failure to produce effective motor output^2,15,28^. These models embedded M1 and other gait-related structures in an “inhibited” or “hypoactive” state^33^ and indeed, we found that M1 was less excitable such that the insufficient “facilitation” of M1 excitability paralleled the freeze. From the present experiment, however, we cannot infer if M1 was actively inhibited or lacked afferent input (or both). This distinction is pathophysiologically meaningful since inhibition versus facilitation of M1 rely on different intracortical circuits and neurotransmitters, which both reflect separate active processes^34,35^. We found that excitability during freezing did not differ significantly from excitability during the transition. In fact, M1 excitability was lowest overall at relaxation during the transition – when motor output was still intact. Motor arrest during freezing, however, occurred involuntarily, suggesting that the abnormally excited M1 may indeed result from activation failure (lack of excitatory afferences) rather than active inhibition. Even though this observation is based on a relatively small sample, we believe it is still worth noting since it highlights the importance of the underlying movement intention (voluntary vs. involuntary motor stops) and the reliance on the cognitive control network to compensate for deficiencies in internal movement generation and motor automaticity^15^. Nonetheless, it remains for future studies to investigate inhibitory plasticity measures during a freeze.

Finally, our findings parallel clinical observations made on freezing of gait, in which TMS protocols increasing M1 excitability have led to an improvement in symptoms, i.e., 10 Hz repetitive TMS of M1^36^, or combined stimulation of the dorsolateral prefrontal cortex and M1 with anodal transcranial direct current stimulation^37^ and further corroborate the notion of an hypoactive M1 during freezing.

There are a few points to consider when interpreting our results. First, the presented data of our pilot study is based on a small sample size, which naturally increases variability and may limit statistical power. Hence, we used a statistical approach focusing not on individual patients but on the pooled number of evoked MEPs per experimental condition. This enabled us to collect sufficient data (regular tapping asc (447 MEPs)/desc (425 MEPs), transition asc (58 MEPs)/desc (78 MEPs), freezing (59 MEPs)) to perform meaningful analyses.

Second, as healthy controls do not freeze, we did not add a healthy control group, even though the logical foundation of our experiment is based on available literature on repetitive movements in health. We know from healthy subjects, for example, that MEP size is modulated along cyclic upper and lower limb movements^23,24^ and combined TMS-EEG studies showed that beta-band desynchronization over the sensorimotor area related to increased M1 excitability and greater MEP size^20,21,38^. In PD patients, our work^8^ and the work of other groups^16^ found that during repetitive finger tapping beta activity modulation was preserved and modulated along the tap cycle in a similar fashion albeit differences have been observed regarding peak beta amplitudes^16–19^. Thus, we concluded that modulation of M1 excitability relative to the tap cycle would be preserved in PD patients, and this is indeed one finding of the present study.

Third, we chose to study patients in the off-medication state only as it allowed to observe the genuine disease-related pathophysiology of freezing and L-Dopa withdrawal significantly increases freezing likelihood^39^. On-state freezing, on the other hand, would have provided heterogeneity in terms of L-Dopa resistant freezing vs. paradoxical on-freezes, both of which are considered different phenomena^40^. Also, previous TMS experiments found no meaningful differences in MEP amplitudes (during isometric contraction) before and after L-Dopa intake^25,26,41,42^ and particularly during finger tapping, cortical beta band modulations did not differ between medication on- and off-states in an EEG experiment with PD patients^43^.

In conclusion, freezing reflects an episodic event of ineffective motor output. Our findings suggest that the Parkinsonian M1 is impaired in increasing excitability during a freeze and, critically, already during the transition phase preceding a freeze. Therapeutic interventions modulating M1 should be investigated as a treatment approach to ameliorate freezing phenomena in Parkinson’s disease.

## Methods

### Patients

We studied eleven right-handed patients with akinetic-rigid iPD and clinically verified FoG^44^ (Table 1). Exclusion criteria were a Mini-Mental State Examination score <22/30, TMS safety concerns^45^, and neurological (ataxia, spasticity, epilepsy) or psychiatric disorders (major depression, substance abuse) that would impact interpretability, performance, and/or compliance. Patients participated with written informed consent and the study protocol followed the Declaration of Helsinki, which was approved by the ethics committee of the University of Tübingen (protocol number: 916/2018BO1).

### Study design

Patients were investigated during their “medication off” state having withdrawn overnight from their dopaminergic medication. Preparation included the mounting of the EMG electrodes, identification of the individual motor hotspot^46^, and determination of the individual active motor threshold (aMT) following standardized procedures published elsewhere^47^. The EMG was recorded from the right first dorsal interosseus (FDI) in the belly-tendon montage (5 kHz sampling rate, 0.16 Hz–1.25 kHz bandpass filter, 24-bit amplifier). Patients placed their right index finger on a force sensor and were instructed to perform internally generated taps as fast and accurately as possible up to a 2 N peak maximum while keeping the finger in permanent contact with the sensor. Real-time visual feedback was provided on tapping force and accuracy. A tapping block lasted 30 s, followed by a 20 s pause to prevent fatigue. Overall, one session included ten blocks of continuous, self-paced tapping or more, if possible.

**Fig. 4:**
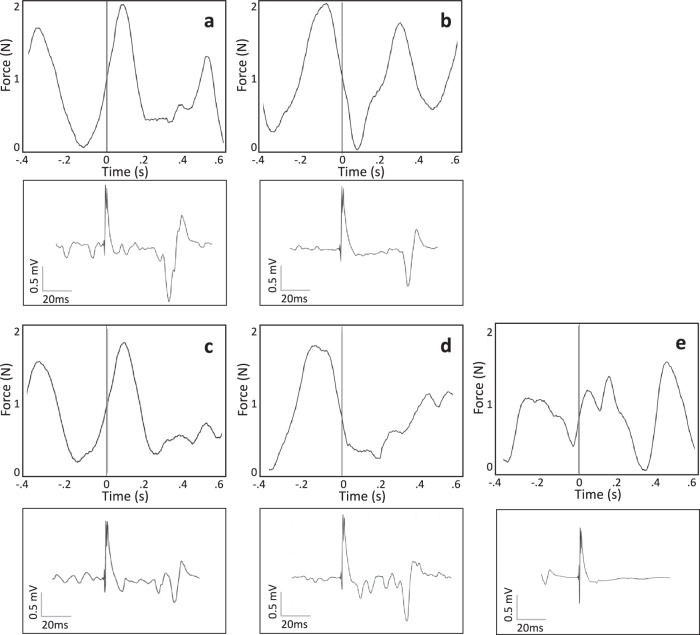
Tapping sections with TMS pulses and corresponding MEPs (exemplary data from one patient). Top panels show sections of kinematic data, that is, continuous tapping (Force, (N)) over time (s) and the time point of the TMS pulse (grey vertical lines). Bottom panels show the corresponding MEP (Amplitude, (µV); 20–40 ms after TMS pulse) recorded over the right first dorsal interosseus (rFDI). For the sake of clarity, different time scales have been chosen for kinematic and MEP panels, but overlay at time ‘0’, i.e. TMS pulse. **a** regular tapping ascending (rTasc), **b** regular tapping descending (rTdesc), **c** transition period ascending (TRANSasc), **d** transition period descending (TRANSdesc), **e** freezing (ULF).

Single-pulse TMS was applied over the hand representation of the left M1 using a figure-of-eight coil (Magstim D70, 70 mm winding; Magstim Ltd., UK) at 120% aMT stimulus intensity^46^. A custom-made real-time signal processing algorithm based on Simulink Real-Time (Version R2016a, MathWorks Inc., USA) was used to trigger single TMS pulses in response to specific pressure sensor force conditions during the tap cycle. We phase-locked the pulses to 25% (ascending) and 75% (descending) of the tap cycle (NeurOne Tesla with Digital-Out Option, Bittium, Finland) in randomized order (Fig. 4). An interstimulus interval of ≥2.5 s was maintained to prevent induction of neural plasticity from repetitive stimulation^48,49^. Transitions or freezes were thus only hit by chance as a reliable real-time prediction based on the kinematic time series has not yet been established for these time segments. Transition segments could only be identified in post-hoc analysis.

### Data segmentation and processing

Preprocessing was done with FieldTrip open-source toolbox^50^ and customized MATLAB® scripts (Mathworks Ltd, USA, R2017a). Each patient’s kinematic data was segmented into single taps and ULF episodes using MATLAB function ‘*findpeaks.m*’. One tap cycle consisted of a downward press (trough to peak) and an upward release (peak to trough). A trough-peak-trough section was considered a full tap if it lasted <2 s and if its amplitude deflection from trough-to-peak and peak-to-trough was ≥1 N (50%)^8^. We then calculated mean tapping frequencies (per patient, overall) and average EMG activity (freezing, regular tapping). ULF episodes were detected visually based on the following criteria: *(i)* reduction of amplitude deflection >50%, *(ii)* Freezing Index (FI) > 1, and *(iii)* duration ≥0.5 s^11,51^.

Peak-to-peak MEPs were extracted from the EMG signal 20–40 ms after the TMS pulse and stored for analysis if >200 µV^47^. Lower amplitudes were set to 0 µV and considered ‘no MEP’ for statistical analyses. The mean MEP size per individual and tapping block served as reference for normalization to account for intra- and interpersonal drifts in cortical excitability^52^. MEPs were classified as either ‘ascending’ or ‘descending’ during regular tapping and in the transition period, which was taken as the last three taps preceding a freeze^8^. MEPs during freezing episodes were not divided into ascending or descending due to the lack of effective motor output when freezing.

It is known that force levels and background EMG activity at or just before single-pulse TMS may influence MEP size^22,53–55^. Therefore, we deliberately chose the right FDI muscle to record MEPs due to its partial agonist activity during finger tapping to reduce the magnitude of these influences.

### Statistical analysis

This piloting study aimed to investigate real-time cortical excitability in PD patients during repetitive finger movements, transitions and freezing. We decided on an exploratory strategy, as *(i)* recording time and adherence of PD patients in “medication off” was limited and *(ii)* the number of freezes and transitions that could be recorded in the laboratory setting and with it the number of MEPs evoked per condition could not be predicted. This also meant that we planned to perform non-parametric pairwise comparisons between contrasts of interest as introduced above, i.e. we had neurophysiological hypothesis as introduced above comparing regular ascending vs. descending, regular ascending vs. ULF, transition ascending vs descending, and transition ascending vs. ULF.

Further, we used a linear multiple regression model to predict MEP size from different independent variables, i) behavioural state (regular tapping ascending, regular tapping descending, freezing), ii) background EMG activity (rooted mean square 10–110 ms prior to stimulation) and force at the time of stimulation. MEP size was treated as dependent outcome variable to ensure that EMG activity and MEP sizes were not linearly related^23^.

Statistical analyses were performed with SPSS® version 25 (IBM), Prism 8 (GraphPad), and Matlab® (Mathworks 2017a Ltd, USA); graphs were created with Prism 8. MEP amplitudes were not normally distributed (*Shapiro-Wilk* test) and thus all statistical tests were two-tailed nonparametric (*Mann-Whitney* U, *Wilcoxon* signed ranks test, *Spearman’s* rho), and a *p-value* of < 0.05 was considered statistically significant. MEPs are presented as percent (%) change from the block average.

### Reporting summary

Further information on research design is available in the Nature Research Reporting Summary linked to this article.

## Supplementary information

Reporting Summary

## Data Availability

Data related to the analysis and findings of this study can be provided to interested researchers upon reasonable request from the principal authors of this study (Marlene Topka and Daniel Weiss). Data are locally stored on the archiving system of the Hertie-Institute for Clinical Brain Research. Since the MEP data and clinical metadata of this study stem from human beings, we consider controlled access to these data under privacy, ethical, and legal issues.

## Code availability

Code related to the analysis and findings of this study can be provided to interested researchers upon reasonable request from the principal authors of this study (Marlene Topka and Daniel Weiss). Code is locally stored on the archiving system of the Hertie-Institute for Clinical Brain Research.
