# Supplementary material for: Motor cortex excitability is reduced during freezing of upper limb movement in Parkinson’s disease
Source: NPJ Parkinsons Dis. 2022 Nov 23;8:161. doi: 10.1038/s41531-022-00420-w (PMC9691624; doi:10.1038/s41531-022-00420-w)
Supplement: Supplementary file 1 — Reporting Summary [file 41531_2022_420_MOESM1_ESM.pdf]

nature portfolio

Topka et al
NPJPARKD-01133R
Corresponding author(s): Correspondence Daniel Weiss

Last updated by author(s): Jun 30, 2022

Reporting Summary

Nature Portfolio wishes to improve the reproducibility of the work that we publish. This form provides structure for consistency and transparency in reporting. For further information on Nature Portfolio policies, see our Editorial Policies and the Editorial Policy Checklist.

Statistics

For all statistical analyses, confirm that the following items are present in the figure legend, table legend, main text, or Methods section.

n/a	Confirmed	
☐	☒	The exact sample size (n) for each experimental group/condition, given as a discrete number and unit of measurement
☐	☒	A statement on whether measurements were taken from distinct samples or whether the same sample was measured repeatedly
☐	☒	The statistical test(s) used AND whether they are one- or two-sided<br>*Only common tests should be described solely by name; describe more complex techniques in the Methods section.*
☒	☐	A description of all covariates tested
☐	☒	A description of any assumptions or corrections, such as tests of normality and adjustment for multiple comparisons
☐	☒	A full description of the statistical parameters including central tendency (e.g. means) or other basic estimates (e.g. regression coefficient) AND variation (e.g. standard deviation) or associated estimates of uncertainty (e.g. confidence intervals)
☐	☒	For null hypothesis testing, the test statistic (e.g. F, t, r) with confidence intervals, effect sizes, degrees of freedom and P value noted<br>*Give P values as exact values whenever suitable.*
☒	☐	For Bayesian analysis, information on the choice of priors and Markov chain Monte Carlo settings
☒	☐	For hierarchical and complex designs, identification of the appropriate level for tests and full reporting of outcomes
☒	☐	Estimates of effect sizes (e.g. Cohen's d, Pearson's r), indicating how they were calculated

Our web collection on statistics for biologists contains articles on many of the points above.

Software and code

Policy information about availability of computer code

Data collection	NeurOne Tesla with Digital-Out Option, Bittium, Finland; Simulink Real-Time, FieldTrip open-source toolbox, and customized MATLAB® scripts
Data analysis	SPSS version 25 (IBM), Prism8 (GraphPad), Matlab (Mathworks 2017a Ltd. USA)

For manuscripts utilizing custom algorithms or software that are central to the research but not yet described in published literature, software must be made available to editors and reviewers. We strongly encourage code deposition in a community repository (e.g. GitHub). See the Nature Portfolio guidelines for submitting code & software for further information.

Data

Policy information about availability of data

All manuscripts must include a data availability statement. This statement should provide the following information, where applicable:

- Accession codes, unique identifiers, or web links for publicly available datasets
- A description of any restrictions on data availability
- For clinical datasets or third party data, please ensure that the statement adheres to our policy

Raw data were generated at the University of Tuebingen. Derived data supporting the findings of this study are available upon reasonable request by the corresponding author Daniel Weiss

Human research participants

Policy information about studies involving human research participants and Sex and Gender in Research.

Reporting on sex and gender	No differentiation between sex and/or gender was made.
Population characteristics	See above.
Recruitment	Participants were recruited either during in-patient stays or out-patient visits.
Ethics oversight	Ethics committee of the University of Tuebingen, Germany, protocol number 916/2018BO1

Note that full information on the approval of the study protocol must also be provided in the manuscript.

Field-specific reporting

Please select the one below that is the best fit for your research. If you are not sure, read the appropriate sections before making your selection.

☒ Life sciences ☐ Behavioural & social sciences ☐ Ecological, evolutionary & environmental sciences

For a reference copy of the document with all sections, see nature.com/documents/nr-reporting-summary-flat.pdf

Life sciences study design

All studies must disclose on these points even when the disclosure is negative.

Sample size	Sample size of 11 patients with MEPS(condition): regular tapping asc (447) / desc (425), transition asc (58) / desc (78), freezing (59).
Data exclusions	No data were excluded from analyses.
Replication	Pilot study. Data have not yet been replicated.
Randomization	No randomization, same protocol for each participant.
Blinding	No blinding. Freezing episodes were detected visually by defined well-established objective criteria and MEP sizes were determined automatically using a Matlab algorithm.

Reporting for specific materials, systems and methods

We require information from authors about some types of materials, experimental systems and methods used in many studies. Here, indicate whether each material, system or method listed is relevant to your study. If you are not sure if a list item applies to your research, read the appropriate section before selecting a response.

Materials & experimental systems

n/a	Involved in the study	
☒	☐	Antibodies
☒	☐	Eukaryotic cell lines
☒	☐	Palaeontology and archaeology
☒	☐	Animals and other organisms
☐	☒	Clinical data
☒	☐	Dual use research of concern

Methods

n/a	Involved in the study	
☒	☐	ChIP-seq
☒	☐	Flow cytometry
☒	☐	MRI-based neuroimaging

Clinical data

Policy information about clinical studies

All manuscripts should comply with the ICMJE guidelines for publication of clinical research and a completed CONSORT checklist must be included with all submissions.

Clinical trial registration	n.a.
Study protocol	no clinical trial, neurophysiological study in human beeing, Parkinsons disease patients

March 2021

Data collection	clinical descriptives (clinical scores) were obtained.
Outcomes	no clinical endpoint in this study, neurophysiological measures (MEP size)
